# Prevalence of *Schistosoma mansoni* and *S. haematobium* in Snail Intermediate Hosts in Africa: A Systematic Review and Meta-analysis

**DOI:** 10.1155/2020/8850840

**Published:** 2020-09-07

**Authors:** Tamirat Hailegebriel, Endalkachew Nibret, Abaineh Munshea

**Affiliations:** Department of Biology, College of Science, Bahir Dar University, Bahir Dar, Ethiopia

## Abstract

**Background:**

Schistosomiasis is caused by *Schistosoma mansoni* and *S. haematobium* in Africa. These schistosome parasites use freshwater snail intermediate hosts to complete their lifecycle. Varied prevalence rates of these parasites in the snail intermediate hosts were reported from several African countries, but there were no summarized data for policymakers. Therefore, this study was aimed to systematically summarize the prevalence and geographical distribution of *S. mansoni* and *S. haematobium* among freshwater snails in Africa.

**Methods:**

Literature search was carried out from PubMed, Science Direct, and Scopus which reported the prevalence of *S. mansoni* and *S. haematobium* among freshwater snails in Africa. The pooled prevalence was determined using a random-effect model, while heterogeneities between studies were evaluated by *I*^2^ test. The meta-analyses were conducted using Stata software, metan command.

**Results:**

A total of 273,643 snails were examined for the presence of *S. mansoni* and *S. haematobium* cercaria in the eligible studies. The pooled prevalence of schistosome cercaria among freshwater snails was 5.5% (95% CI: 4.9–6.1%). The pooled prevalence of *S. mansoni* and *S. haematobium* cercaria was 5.6% (95% CI: 4.9–6.3%) and 5.2% (95% CI: 4.6–5.7%), respectively. The highest pooled prevalence was observed from Nigeria (19.0%; 95% CI: 12.7–25.3%), while the lowest prevalence was reported from Chad (0.05%; 95% CI: 0.03–0.13). Higher prevalence of schistosome cercaria was observed from *Bulinus globosus* (12.3%; 95% CI: 6.2–18.3%) followed by *Biomphalaria sudanica* (6.7%; 95% CI: 4.5–9.0%) and *Biomphalaria pfeifferi* (5.1%; 95% CI: 4.1–6.2%). The pooled prevalence of schistosome cercaria obtained using PCR was 26.7% in contrast to 4.5% obtained by shedding cercariae.

**Conclusion:**

This study revealed that nearly 6% of freshwater snails in Africa were infected by either *S. haematobium* or *S. mansoni*. The high prevalence of schistosomes among freshwater snails highlights the importance of appropriate snail control strategies in Africa.

## 1. Introduction

Schistosomiasis is one of the neglected tropical diseases (NTD) endemic in 78 countries and infects more than 229 million peoples in tropical and subtropical regions [1]. More than 90% of these cases are concentrated in Africa [2, 3]. The burden of the disease is even more severe in sub-Saharan Africa. Poor environmental sanitation and suitability of the climate conditions for snail intermediate host contribute to the high endemicity of the region. Schistosomiasis ranks second next to malaria from parasitic infection in terms of socioeconomic and health impact in tropics [4].

Human schistosomiasis is caused by *Schistosoma mansoni, S. haematobium, S. japonicum, S. intercalatum, S. mekongi, S. malayensis*, and *S. guineensis* [5–7]. Among these species, *S. mansoni, S. haematobium*, and *S. japonicum* are the major causes of human schistosomiasis globally [8]. *Schistosoma mansoni* and *S. haematobium* are widely distributed and the dominant cause of human schistosomiasis in Africa [5]. The endemicity of the disease in the region is linked with the availability of freshwater snail intermediate hosts.

About 350 species of freshwater snails are known to be medically or veterinary important [9]. Among these diverse snails, *Biomphalaria, Bulinus*, and *Oncomelania* snails [4] are the dominant snail genera that are involved in the transmission of human schistosomiasis. The *Biomphalaria genus* consists of *B. pfeifferi, B. glabrata, B. sudanica, B. straminea, B. tenagophila, B. alexandarina*, and *B. choanomphala* [10]. *Biomphalaria* snails serve as the intermediate host for *S. mansoni*, which is responsible for intestinal and hepatic schistosomiasis. *Biomphalaria pfeifferi* is the most common and widely distributed snail intermediate host for *S. mansoni* in Africa.

*Bulinus* consists of 37 recognized species, which is grouped mainly into four species groups, namely, *Bulinus africanus, B. forskalii, B. truncates/tropicus,* and *B. reticulatus* [10, 11]. *Bulinus* snails serve as intermediate hosts for *S. haematobium*, which is responsible for urinary schistosomiasis. *Oncomelania* snails consist of only a few species mainly reported from Asia. The most common snail intermediate host for *S. japonicum* is *Oncomelania hupensis,* which is found in China, the Philippines, Indonesia, and also Japan [12].

The prevalence of human schistosomiasis is varied greatly in African countries depending on the level of environmental sanitation and the suitability of the area for the snail intermediate hosts, as well as the type of snail in the area. Similarly, the prevalence of schistosomes cercaria in snail intermediate hosts is varied in different locations within the same country and also from country to country in Africa. Several epidemiological studies are available on the types and prevalence of human infecting schistosomes among snail intermediate hosts in Africa. However, up to this time, there has not been any single estimate of the prevalence of *S. mansoni* and *S. haematobium* in snail intermediate hosts in Africa that could be used by African policymakers and international organizations working on the prevention and control of schistosomiasis in the continent. Therefore, this study aimed to provide summarized data on the prevalence and geographical variations of *S. mansoni* and *S. haematobium* cercaria among freshwater snails in Africa.

## 2. Methods

### 2.1. Search Strategies

Relevant literature was systematically searched from online public databases (PubMed Central, Science Direct, and Scopus) using the following key-words: “Schistosomiasis” OR “*S. mansoni*” OR “*S. haematobium*” OR “parasitological study” OR “schistosome intermediate host” OR “freshwater snails” OR “malacological survey” OR “*Biomphalaria* snails” OR “*Bulinus* snails” AND “Africa”. The systematic review and selection of relevant literature were conducted according to PRISMA (Preferred Reporting Items for Systematic Reviews and Meta-analysis) guidelines [13] (Table S1).

### 2.2. Inclusion and Exclusion Criteria

Literature published in English language from 1979 to June 2020 were extracted from online public databases. Original articles reporting the prevalence of human schistosome cercariae in freshwater snails in African countries were included in the analysis. The eligibility for the inclusion of a study in our analysis had to fulfill the following criteria: (a) it was published in English, (b) the study was carried out in Africa, (c) the number of examined and infected snails with either *S. mansoni* or *S. haematobium* were clearly stated, and (d) snail species were identified at least to a genus level. Studies that reported nonhuman schistosome and other trematodes species were excluded from the analysis. Besides, review articles and meta-analysis were excluded from the analysis.

### 2.3. Data Extraction Protocol

The data extraction protocol was prepared and evaluated by all authors. From each published article, we extracted the following information: author information, year of publication, study country, snail species, number of snails (collected, examined and infected), the prevalence of snail infection, the type of schistosomes reported, and methods of schistosome's detection.

### 2.4. Quality of Individual Study and Assessment of Bias

The quality of studies included in the meta-analysis was assessed by using the Newcastle–Ottawa quality assessment scale (NOS) proposed by Wells et al. [14] (Text S1). The quality assessment tool consists of three parts. First, the selection of study groups graded on a scale containing five stars; mainly deals with methodological qualities of individual study. Second, comparability of groups graded on a scale containing two stars; deals with comparability of studies based on design and analysis. Third, outcomes graded on a scale containing three stars, mainly focused on the assessment of the outcome and statistical analysis. Two authors (TH and EN) independently assessed the quality of individual study, and disagreement was solved by a discussion with the third author (AM). The overall quality of the individual study was categorized as high quality (≥8 stars), moderate quality (6-7 stars), and low quality (≤5 stars) by the total number stars obtained as described elsewhere [15].

### 2.5. Publication Bias across Studies

The risks of publication bias across studies were assessed using funnel plot symmetry qualitatively. Egger's and Begg's test were used to determine the presence of publication bias across studies quantitatively.

### 2.6. Data Analysis

We used a forest plot to estimate the overall pooled effect size with their 95% confidence interval (CI). The heterogeneity among studies used for this meta-analysis was evaluated using the *I*^2^ test [16]. An *I*^2^- value lower than 25%, between 25% and 50%, and above 50% was regarded as low, moderate, and high heterogeneity, respectively [17]. Because of the high heterogeneity observed among the studies included in the meta-analysis, we used a random-effect model at 95% CI. To sort out the cause of heterogeneity, we used a subgroup analysis, sensitivity test, and meta-regression analysis. The data analysis was conducted using Stata software (version 14, STATA Corp College Station, TX), “metan” command.

## 3. Results

### 3.1. Search Results and Eligible Studies

**Figure 1:**
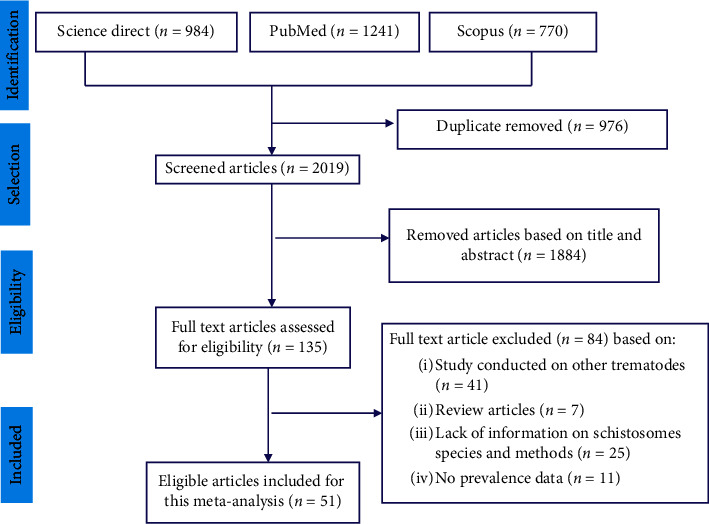
PRISMA flow diagram of for the inclusion of studies on the prevalence of *S. mansoni* and *S. haematobium* in freshwater snails in Africa.

A total of 2,995 relevant studies were screened from online public databases. Out of these studies, 976 articles were removed due to duplications while 1884 articles were excluded based on title and abstract screening. The remaining 135 full-text articles were assessed for eligibility. Of these, a total of 84 articles were excluded from the analysis based on specific exclusion criteria, and the remaining 51 articles were selected for this meta-analysis (Figure 1).

### 3.2. Characteristics of Subjects in the Eligible Studies

The eligible articles were obtained from 17 African countries: Angola [18], Benin [19], Burkina Faso [20], Burundi [21], Chad [22], Côte d'Ivoire [23, 24], Egypt [25–30], Ethiopia [31–36], Kenya [37–39], Mali [40, 41], Niger [42, 43], Nigeria [44–54], Senegal [55, 56], Sudan [57], Tanzania [58–63], Uganda [64–67], and Zimbabwe [68]. Unfortunately, there were no studies from other African countries that fulfilled the inclusion criteria. Characteristics of the eligible article to this meta-analysis are presented in Table 1.

### 3.3. Risk of Bias within Studies

The Newcastle–Ottawa quality assessment scale indicated that there was no bias within studies. The individual study included in this review was moderate to high-quality score as indicated in Table 1.

### 3.4. Prevalence of *S. mansoni* and *S. haematobium* Cercaria Among Freshwater Snails

**Figure 2:**
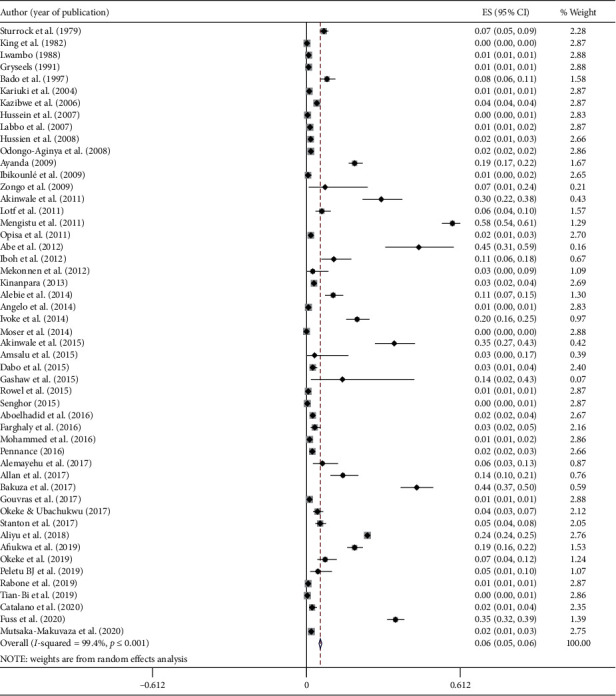
Forest plot diagram showing the prevalence of human infecting schistosomes (*S. mansoni* and *S. haematobium*) examined in snails in Africa. Each square represent effect size (ES) of individual studies, and the horizontal line represents the 95% CI. The diamond indicates the pooled effect and the vertical dash lines indicate the overall estimate.

A total of 273, 643 snails from *Biomphalaria* and *Bulinus* genera were examined for the presence of *S. mansoni* and *S. haematobium* cercaria in the 51 eligible studies, respectively (Table 2). Out of these snails, 8,682 of them were infected by either *S. mansoni* or *S. haematobium*. The prevalence of schistosome cercaria in the individual study ranged from 0.05% to 58.03% with substantial heterogeneity across studies within and across countries. The pooled prevalence of schistosome cercaria among freshwater snails was 5.5% (95% CI: 4.9–6.1%, *I*^2^ = 99.4%, and *p* < 0.001) (Figure 2).

### 3.5. Subgroup Analysis

**Figure 3:**
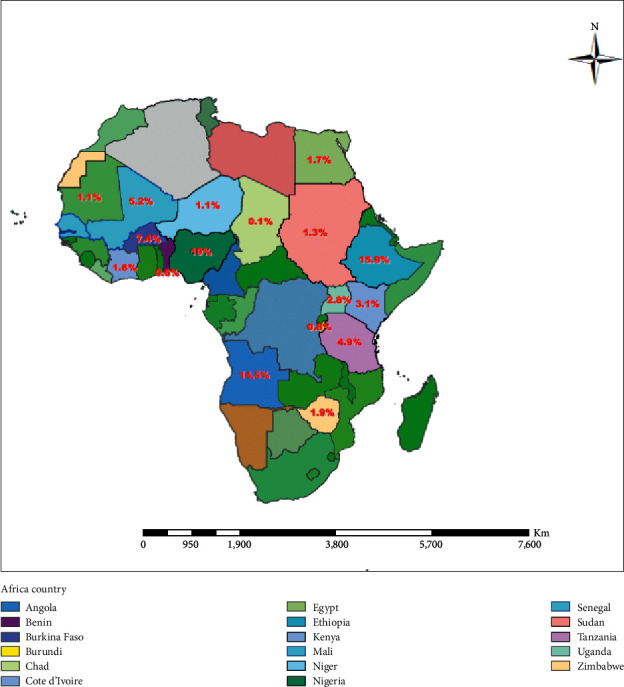
Geographical distribution and pooled prevalence of *S. mansoni* and *S. haematobium* among *Biomphalaria* and *Bulinus* snails, respectively, in African countries.

**Figure 4:**
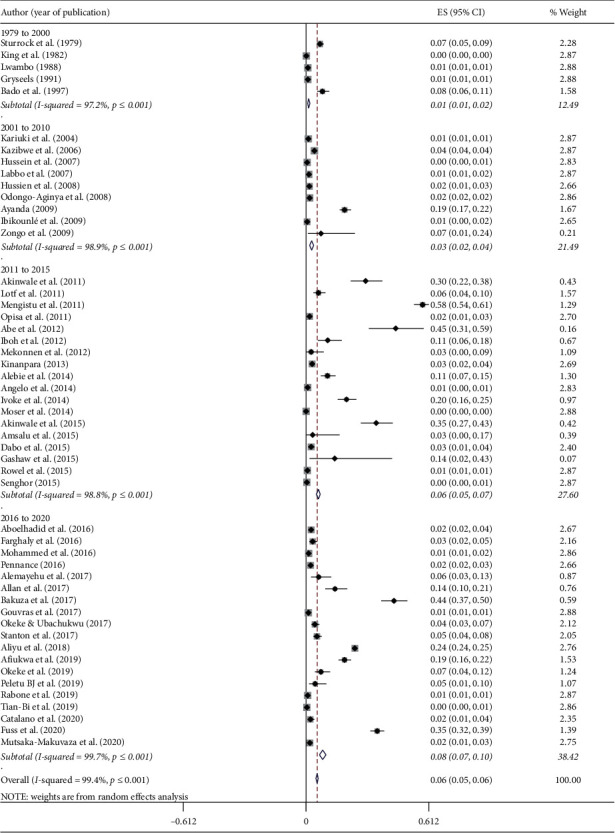
The pooled prevalence of schistosome cercaria among freshwater snails in Africa based on year of publication.

The highest pooled prevalence of schistosome cercaria was observed among freshwater snails from Nigeria (19.0%; 95% CI: 12.7–25.3%), followed by Ethiopia (15.9%; 95% CI: −5.9–37.5%), Mali (5.2%; 95% CI: -0.3–10.7%), and Tanzania (4.9%; 95% CI: 3.8–6.0%) (Figure 3). We categorized the years of studies into four groups: before 2000, 2001 to 2010, 2011 to 2015, and 2016 to June 2020 to assess the trends on the prevalence of schistosome cercaria in freshwater snails. The pooled prevalence of schistosome cercaria among freshwater snails in years before 2000, 2001–2010, 2011–2015, and 2016–2020 was 1.3% (95% CI: 0.8–1.8%), 2.8% (95% CI: 1.8–3.8%), 6.1% (95% CI: 5.2–7%), and 8.3% (95% CI: 6.6–9.9%), respectively, in Africa (Figure 4).

**Figure 5:**
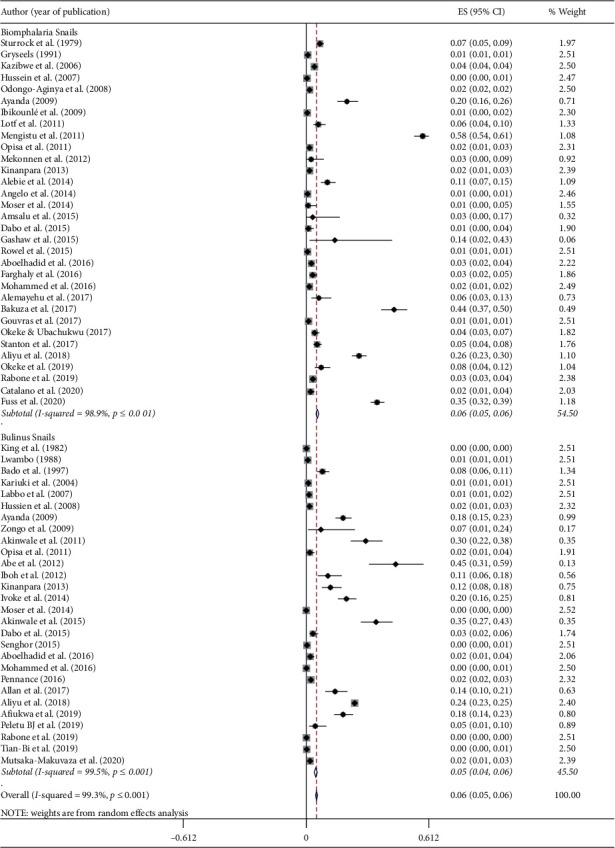
Forest plot diagram showing the estimated effect size of *S. mansoni* in *Biomphalaria* snails and *S. haematobium Bulinus* snail in Africa.

This meta-analysis targets the two most common and widely distributed *Schistosoma* species (*S. mansoni* and *S. haematobium*) in the continent. *Biomphalaria* and *Bulinus* snails were the intermediate hosts for *S. mansoni* and *S. haematobium,* respectively. The pooled prevalence of *S. mansoni* cercaria in *Biomphalaria* snails was 5.6% (95% CI: 4.9–6.3%) while the pooled prevalence of *S. haematobium* cercaria in *Bulinus* snails was 5.2% (95% CI: 4.7–5.7%) (Figure 5).

The pooled prevalence of *S. mansoni* and *S. haematobium* among freshwater snails was varied from country to country. The highest pooled prevalence of *S. mansoni* among *Biomphalaria* snails was observed from Tanzania (16.6%) followed by Ethiopia (15.9%) and Nigeria (14.5%) (Figure S1). On the contrary, the highest pooled prevalence of *S. haematobium* among *Bulinus* snail was observed from Nigeria (19.6%) followed by Angola (14.5%) and Côte d'Ivoire (9.6%) (Figure S2).

Twelve snail species from *Biomphalaria* and *Bulinus* snails were reported in the eligible articles used for this meta-analysis (Table 2). Among these species, *Biomphalaria pfeifferi* was the most common snail species and reported from 19 studies (37.3%) from the total 51 studies included in this study. The pooled prevalence of *S. mansoni* cercaria was 5.1% (95% CI: 4.1–6.2%) among *B. pfeifferi* snails. *Bulinus* snail, particularly *B. globosus* and *B. truncatus*, were the second and third most reported snails species (reported in 14 and 13 studies, respectively) that serve as an intermediate host for *S. haematobium*. The pooled prevalence of *S. haematobium* cercaria was 12.3% (95% CI: 6.2–18.3%) and 5.8% (95% CI: 4.4–7.2%) in *B. globosus* and *B. truncatus* snails, respectively (Table 2).

**Figure 6:**
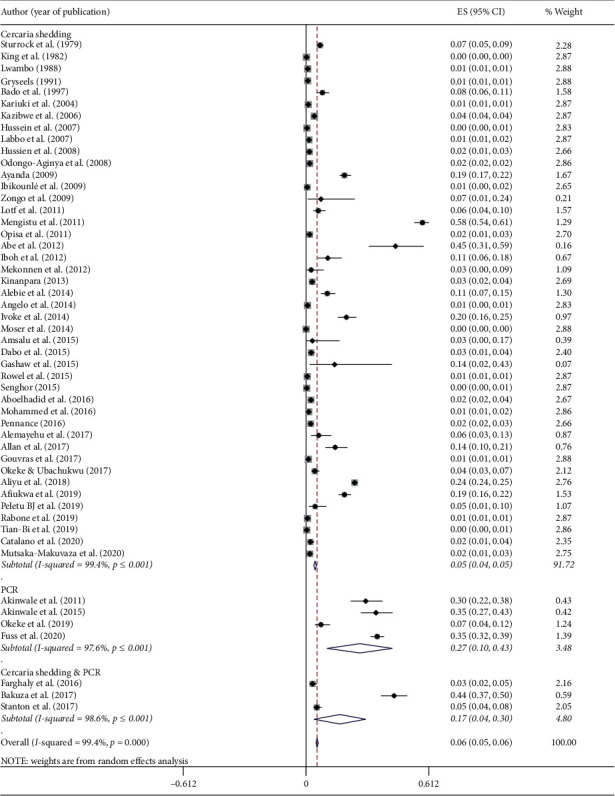
Forest plot diagram showing the pooled prevalences of *S. mansoni* and *S. haematobium* among freshwater snails examined by cercarial shedding and PCR.

The studies included in this meta-analysis used shedding of cercariae and PCR-based detection of schistosomes from snail tissue. The pooled prevalence of schistosomes obtained by shedding of cercariae was 4.5% (95% CI: 3.9–5.1%) in contrast to 26.7% (95% CI: 10.5–43.0%) obtained by PCR techniques (Figure 6).

### 3.6. Publication Bias across Studies

**Figure 7:**
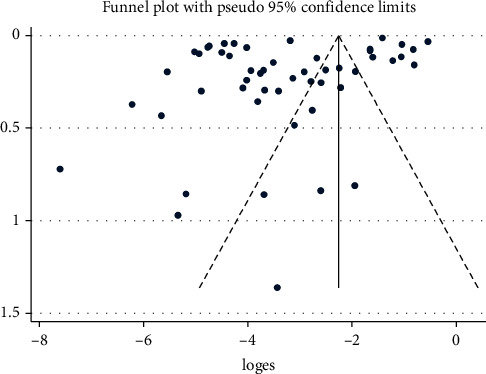
Funnel plot with 95% confidence limit showing publication bias across studies on the prevalence of *S. mansoni* and *S. haematobium* among freshwater snails of Africa.

The funnel plot symmetry demonstrates the presence of publication bias among studies included in this meta-analysis (Figure 7). Similarly, Egger's test results (*p*=0.02) and Begg's test (*p*=407) confirm the presence of publication bias among studies.

### 3.7. Metaregression Analysis and Sensitivity Test

There were clear heterogeneities across studies included in this meta-analysis. We performed a meta-regression analysis to identify the sources of heterogeneity across studies. The metaregression analysis showed that methods of schistosome detection from snails (regression coefficient: 2.55, 95% CI, 1.17–5.54, *p*=0.02) might be the source of heterogeneity. The country of study (regression coefficient: 0.99, 95% CI, 0.89–1.11, *p*=0.97), years of publication (regression coefficient: 1.41, 95% CI, 0.91–2.18, *p*=0.12), snail genus (regression coefficient: 1.19, 95% CI, 0.56–2.55, *p*=0.65), and snail species (regression coefficient: 0.92, 95% CI, 0.8 *p*=0.65*p*=0.14) did not contribute for the heterogeneity. Besides, a sensitivity analysis was performed by recalculating the pooled prevalence by sequentially removing one-by-one to assess the effect of individual studies to overall effect. The pooled prevalence remained stable, and the result was not driven by individual studies included in the meta-analysis.

## 4. Discussion

Schistosomiasis is the second leading cause of infectious diseases next to malaria in Africa. Despite intensive efforts to tackle schistosomiasis, the prevalence is still unacceptably high in many African countries. The control and prevention strategies mainly rely on treatment of infected cases and mass drug administration of school-aged children. In many African countries, snail control strategies are not routinely implemented and sometimes considered as old-fashion approaches [70] despite their vital contributions to the elimination of schistosomes witnessed from Japan, Iran, and Tunisia [71–73]. In addition, enormous progresses have been observed in the elimination program from Morocco, Oman, Lebanon, and Caribbean Islands [73, 74]. The intensity and prevalence of schistosomes' infection among freshwater snails are scarce from many African countries. Summarized information about the prevalence of schistosomes among snail intermediate hosts is important for policymakers to give better attention to snail control strategies in Africa.

The overall pooled prevalence of schistosome cercaria was nearly 6% among freshwater snails in Africa. This finding is slightly lower than 9% reported from freshwater snails in Brazil [75]. In contrast, a lower prevalence of infected snails was observed from Indonesia [76] and Brazil [77]. These differences might be associated with prevalence and intensity of schistosome infection in the community, the level of environmental sanitation, suitability of the climate for the snails, level of existing snail control strategies, level of human exposure to open surface water, methods of schistosome detection, and seasons of snail collection and examination.

The highest pooled prevalence of schistosome cercaria among freshwater snails was observed in Nigeria followed by Ethiopia. In contrast, low prevalences of schistosomes were observed from Benin, Burundi, and Chad. The high prevalence of schistosomes among snail species in Nigeria and Ethiopia might be associated with the high prevalence of schistosomes in the community. The prevalence of schistosomiasis could reach as high as 90% in Ethiopia [78] and 94% in Nigeria [79, 80]. In addition, the difference in the level of environmental sanitation and the suitability of the area for the intermediate host, as well as the types of snail species in the area, may contribute for the difference in infection of snails across countries. Moreover, larger numbers of studies were reported from these two countries. Out of 51 studies, 17 (23.9%) studies included in this review were from the two countries.

Several species of freshwater snails that potentially serve as intermediate hosts for *S. mansoni* and *S. haematobium* have been recently reviewed [81]. *Biomphalaria* and *Bulinus* snails are the common and widely distributed intermediate hosts for schistosomes in Africa. The twelve snail species observed in this review belonged to either *Biomphalaria* or *Bulinus* genus.

*Biomphalaria* snails are well-known intermediate hosts of *S. mansoni* in Africa. This review showed that 5.6% of *Biomphalaria* snails were infected by *S. mansoni* in Africa. The highest pooled prevalence of *S. mansoni* was observed from Tanzania followed by Ethiopia and Nigeria, while low pooled prevalence was reported from Benin, Burundi, and Chad. These reported differences might be associated with the level of environmental sanitation, the abundance of *Biomphalaria* snails, and seasons of snail collection and examination. The highest prevalence of *S. mansoni* among *Biomphalaria* snails was reported during the dry season or just before the beginning of the rain seasons [47, 82].

Five species of *Biomphalaria* snails (*B. pfeifferi, B. sudanica, B. choanomphala*, *B. alexandrina*, and *B. stanleyi*) were included in the eligible articles for this review. As reviewed by Abe et al. [81], *B. pfeifferi, B. sudanica, B. choanomphala*, and *B. alexandrina* were the common intermediate hosts of *S. mansoni* in Africa. The pooled prevalence of *S. mansoni* varied from 1.3% to 6.7% among these snail species.

*Biomphalaria pfeifferi* were the most common snails infected by schistosome parasite. About 40% of the eligible studies included in this meta-analysis reported *B. pfeifferi.* The role of *B. pfeifferi* as an intermediate host of *S. mansoni* varied from country to country. *Biomphalaria pfeifferi* is the sole intermediate host for *S. mansoni* in Côte d'Ivoire [83] and Senegal [84] and the dominant intermediate host in many other African countries [85–88].

*Biomphalaria sudanica* was the second common *Biomphalaria* snails that serve as an intermediate host for *S. mansoni* as observed in this study. *Biomphalaria sudanica* is an intermediate host for *S. mansoni* in Kenya [47] and Tanzania [19, 89]. *Biomphalaria sudanica* is also reported from Ethiopia but limited to around Lake Ziway [90] and Tikur Wuha [87].

*Biomphalaria alexandrina* was the third common intermediate host for *S. mansoni* observed in this study. However, its contribution as an intermediate host for *S. mansoni* is restricted in geographical distribution, mainly reported from Egypt [91–93]. *Biomphalaria choanomphala* was another intermediate host for *Schistosoma* mansoni reported from Uganda [64] and Tanzania [61]. This snail species is widely distributed around Lake Victoria, which is divided among three countries (Kenya, Tanzania, and Uganda). *Biomphalaria choanomphala* is also reported from Kenya [94], but the infection intensity was not determined.

*Bulinus* snail is the common intermediate host for *S. haematobium* in Africa. This study revealed that about 5.2% of *Bulinus* snails were infected with *S. haematobium* in Africa. The highest pooled prevalence of *S. haematobium* infection among *Bulinus* snail was observed from Nigeria followed by Angola and Côte d'Ivoire. In contrast, there was a low prevalence of *S. haematobium* infection among *Bulinus* snails from Chad, Niger, Senegal, and Sudan. These variations might be associated with the difference in the level of endemicity of *S. haematobium* in the countries. A recent review indicated that about one-third of the populations of Nigeria were infected by *S. haematobium* [95, 96]. The higher infection intensity in the population might lead to a high level of environmental contamination that resulted in higher snail infection in Nigeria.

The eligible studies included in this review report seven species of *Bulinus* snails (*B. truncates B. globosus, B. forskalii, B. senegalensis, B. nasutus*, *B. camerunensis*, *and B. umbilicatus)* from African countries. According to Abe et al. [81], *B. truncates, B. africanus, B. forskalii, B. senegalensis*, and *B. camerunensis* were the predominant *Bulinus* snails that serve as an intermediate host for *S. haematobium*.

Among the *Bulinus* snails, *B. globosus* was reported from 14 studies in 7 African countries included in this review. The present study revealed that 12.3% of *B. globosus* was infected by *S. haematobium.* Similarly, high pooled prevalence (18%) of *S. haematobium* among *B. globosus* was recently reported in a meta-analysis from Nigeria [96]. *Bulinus truncatus* are the other important *Bulinus* snails that serve as an intermediate host for *S. haematobium* in Africa. The pooled prevalence of *S. haematobium* was 5.9% among *Bulinus truncatus* snails in Africa. In contrast to our result, 19% prevalence of *S. haematobium* was reported from *B. truncates* snails in Nigeria [31]. Similarly, *B. truncatus* is the predominant intermediate host for *S. haematobium* in Niger [42] and Côte d'Ivoire [23, 24].

Detection of schistosome infection is determined by shedding of cercariae and/or PCR based approaches from snail tissue. There was a significant difference in the pooled prevalence of schistosome results between shedding of cercaria (4.5%) and PCR (26.7%) among snail species in Africa. Similar differences (1.56% vs. 39.8%) are seen in the prevalence of schistosome infection between cercarial shedding and PCR methods among snails as reported from Kenya [97]. This difference is associated with the sensitivity of PCR to detect schistosome infection from snail tissue. Cercarial shedding is suffered by several limitations such as low parasite burden; snails may not shed cercariae during the prepatent period; time-consuming, and labour-intensive [98]. PCR-based detection of schistosome infection from snails is generally rapid, efficient, sensitive, and cost-effective for large-scale detection [99, 100].

Despite the ongoing schistosomiasis control strategies in many African countries, the pooled prevalence of schistosomes among freshwater snails had increased over time from 1.3% (before 2000) to 8.3% between 2016 and 2020. This might be attributed to the large number of epidemiological studies conducted and reported recently. Besides, molecular based detection of schistosome infection from snail tissue received attention in the recent years. These situations may contribute to the increased prevalence of schistosomes among freshwater snails recently.

### 4.1. Limitation of the Study

Although this systematic review generated valuable data on the prevalence of *S. mansoni* and *S. haematobium* among freshwater snails in Africa, it also has limitations. First, information on the prevalence of schistosome cercaria among snail species was not obtained from all African countries. Prevalence data were available only from 17 African countries. The pooled prevalences of schistosomes in this review may not fully represent the prevalence of *S. mansoni* and *S. haematobium* among freshwater snails of Africa. Second, the numbers of published studies were not evenly distributed even in the 17 countries (varied from 1 study to 11 studies in a country). Third, the studies included in this review were published in English, and we did not include studies published in other languages such as French due to language barriers and translation-related challenges. Fourth, most of the studies included in this review used cercarial shedding rather than PCR for the detection of schistosome infection. Cercarial shedding is less sensitive for the detection of schistosomes due to its inherent limitation that may result in low prevalence of schistosomes among infected snails in Africa. The pooled prevalence of schistosome cercaria among freshwater snails of Africa observed in this review might be below the actual infection intensity. Fifth, this review showed that there was high heterogeneity across studies included in this review. These might be associated with study design, seasons of snail collection, method of detection, and variation of endemicity of schistosomes across countries.

## 5. Conclusions

This review showed that nearly 6% of freshwater snails in Africa were infected by either *S. haematobium* or *S. mansoni*. The pooled prevalences of schistosome cercaria among freshwater snails have increased in the recent years in many African countries. The higher and increased trends in the prevalence of schistosomes among freshwater snails highlight the need for appropriate snail control strategies in the region. Policymakers should give better attention about integration of snail control strategies to the ongoing treatment-based prevention of schistosomiasis in Africa.

## Figures and Tables

**Table 1:** Characteristic of the 51 eligible studies and pooled prevalences of *S. mansoni* and *S. haematobium* among freshwater snails in Africa included in this meta-analysis.

Author	Year	Country	Snail species	Snails examined (*n*)	Infected snails					Total		*Schistosoma* species	Quality score
					Cercaria shedding (*n*)	Prevalence (%)	Screened by PCR	Positive (*n*)	Prevalence (%)	Infected snails (*n*)	Prevalence (%)		
Sturrock et al. [39]	1979	Kenya	*B. pfeifferi*	938	65	6.93	—	—	—	65	6.93	*S. mansoni*	7
King et al. [30]	1982	Egypt	*Bullines* spp.	4312	9	0.21	—	—	—	9	0.21	*S. haematobium*	8
Lwambo [60]	1988	Tanzania	*B. nasutus*	17646	156	0.88	—	—	—	156	0.88	*S. haematobium*	9
Gryseels [21]	1991	Burundi	*B. pfeifferi*	29199	249	0.85	—	—	—	249	0.85	*S. mansoni*	9
Bado et al. [40]	1997	Mali	*B. truncatus*	266	-	-	266	22	8.30	22	8.3	*S. haematobium*	8
			*B. globosus*	90	7	7.80	—	—	—	7	7.80	*S. haematobium*	
Kariuki et al. [37]	2004	Kenya	*B. nasutus*	11000	122	1.20	—	—	—	122	1.20	*S. haematobium*	8
Kazibwe et al. [66]	2006	Uganda	*B. stanleyi*	21715	949	4.40	—	—	—	949	4.40	*S. mansoni*	9
			*B. sudanica*	8452	296	3.50	—	—	—	296	3.50	*S. mansoni*	
Hussien et al. [29]	2007	Egypt	*B. alexandrina*	2070	10	0.48	—	—	—	10	0.48	*S. mansoni*	8
Labbo et al. [43]	2007	Niger	*B. forskalii*	9450	5	0.05	—	—	—	5	0.05	*S. haematobium*	8
			*B. truncatus*	27125	509	1.90	—	—	—	509	1.90	*S. haematobium*	
Hussien et al. [25]	2008	Egypt	*B. truncatus*	837	14	1.67	—	—	—	14	1.67	*S. haematobium*	7
Odongo-Aginya et al. [64]	2008	Uganda	*B. choanomphala*	9194	164	1.78	—	—	—	164	1.78	*S. mansoni*	9
			*B. pfeifferi*	4173	75	1.79	—	—	—	75	1.79	*S. mansoni*	
Ayanda [47]	2009	Nigeria	*B. globosus*	392	72	18.37	—	—	—	72	18.37	*S. haematobium*	7
			*B. pfeifferi*	256	52	20.31	—	—	—	52	20.31	*S. mansoni*	
Ibikounlé et al. [19]	2009	Benin	*B. pfeifferi*	357	2	0.56	—	—	—	2	0.56	*S. mansoni*	8
Zongo et al. [20]	2009	Burkina Faso	*B. truncatus*	27	2	7.40	—	—	—	2	7.40	*S. haematobium*	7
Akinwale et al. [53]	2011	Nigeria	*B. truncatus*	138	—	—	138	41	29.70	41	29.70	*S. haematobium*	7
Lotf et al. [26]	2011	Egypt	*B. alexandrina*	277	17	6.10	—	—	—	17	6.10	*S. mansoni*	7
Mengistu et al. [34]	2011	Ethiopia	*Biomphalaria* spp.	560	325	58.00	—	—	—	325	58.00	*S. mansoni*	7
Opisa et al. [38]	2011	Kenya	*B. pfeifferi*	425	7	1.60	—	—	—	7	1.60	*S. mansoni*	9
			*B. sudanica*	407	7	1.70	—	—	—	7	1.70	*S. mansoni*	
			*B. globosus*	227	5	2.20	—	—	—	5	2.20	*S. haematobium*	
Abe et al. [50]	2012	Nigeria	*B. truncatus*	56	25	44.64	—	—	—	25	44.64	*S. haematobium*	7
Iboh et al. [48]	2012	Nigeria	*B. globosus*	120	13	10.80	—	—	—	13	10.80	*S. haematobium*	8
Mekonnen et al. [35]	2012	Ethiopia	*B. pfeifferi*	80	2	2.50	—	—	—	2	2.50	*S. mansoni*	7
Kinanpara [23]	2013	Côte d'Ivoire	*B. globosus*	189	23	12.17	—	—	—	23	12.17	*S. haematobium*	7
			*B pfeifferi*	1409	25	1.77	—	—	—	25	1.77	*S. mansoni*	
Alebie et al. [31]	2014	Ethiopia	*B. pfeifferi*	301	32	10.60	—	—	—	32	10.60	*S. mansoni*	7
Angelo et al. [62]	2014	Tanzania	*B. sudanica*	1470	11	0.75	—	—	—	11	0.75	*S. mansoni*	8
Ivoke et al. [49]	2014	Nigeria	*B. globosus*	308	62	20.10	—	—	—	62	20.10	*S. haematobium*	8
Moser et al. [22]	2014	Chad	*B. truncatus*	4119	1	0.80	—	—	—	1	0.02	*S. haematobium*	8
			*B. pfeifferi*	108	1	0.90	—	—	—	1	0.90	S, mansoni	
Akinwale et al. [54]	2015	Nigeria	*B. globosus*	109	—	—	109	38	34.80	38	34.80	*S. haematobium*	9
			*B. forskalii*	22	—	—	22	8	36.40	8	36.40	*S. haematobium*	
			*B. camerunensis*	7	—	—	7	4	57.00	4	57.00	*S. haematobium*	
			*B. senegalensis*	11	—	—	11	2	18.20	2	18.20	*S. haematobium*	
Amsalu et al. [33]	2015	Ethiopia	*B. pfeifferi*	31	1	3.20	—	—	—	1	3.20	*S. mansoni*	7
Bado et al. [41]	2015	Mali	*B. truncatus*	324	11	3.40	—	—	—	11	3.40	*S. haematobium*	8
			*B. pfeifferi*	189	2	1.10	—	—	—	2	1.10	*S. mansoni*	
Gashaw et al. [32]	2015	Ethiopia	*B. pfeifferi*	14	2	14.30	—	—	—	2	14.30	*S. mansoni*	7
Rowel et al., [67]	2015	Uganda	*Biomphalaria* spp	19355	127	0.70	—	—	—	127	0.70	*S. mansoni*	9
Senghor [55]	2015	Senegal	*B. senegalensis*	7333	8	0.11	—	—	—	8	0.11	*S. haematobium*	9
			*B. umbilicatus*	339	22	6.51	—	—	—	22	6.51	*S. haematobium*	
Aboelhadid et al. [27]	2016	Egypt	*B. alexandrina*	822	22	22.80	—	—	—	22	2.68	*S. mansoni*	7
			*B. truncatus*	423	9	2.10	—	—	—	9	2.10	*S. haematobium*	
Farghaly et al. [28]	2016	Egypt	*B. alexandrina*	400	3	0.8	400	13	3.3	13	3.3	*S. mansoni*	9
Mohammed et al. [57]	2016	Sudam	*B. truncatus*	1403	2	0.10	—	—	—	2	0.10	*S. haematobium*	9
			*B. pfeifferi*	5100	82	1.60	—	—	—	82	1.60	*S. mansoni*	
Pennance [63]	2016	Tanzania	*B. globosus*	1111	26	2.30	—	—	—	26	2.30	*S. haematobium*	8
Alemayehu et al. [36]	2017	Ethiopia	*B. pfeifferi*	111	7	6.30	—	—	—	7	6.30	*S. mansoni*	8
Allan et al. [18]	2017	Angola	*B. globosus*	173	25	14.50	—	—	—	25	14.50	*S. haematobium*	8
Bakuza et al. [69]	2017	Tanzania	*B. pfeifferi*	235	29	12.30	219	103	47.00	103	43.8	*S. mansoni*	8
Gouvras et al. [61]	2017	Tanzania	*B. sudanica*	35,910	439	1.21	—	—	—	439	1.21	*S. mansoni*	7
			*B. choanomphala*	6906	61	0.88	—	—	—	61	0.88	*S. mansoni*	
Okeke and Ubachukwu [52]	2017	Nigeria	*B. pfeifferi*	460	20	4.30	—	—	—	20	4.30	*S. mansoni*	8
Stanton et al. [65]	2017	Uganda	*Biomphalaria* spp.	499	5	1.00	118	22	18.64	27	5.4	*S. mansoni*	8
Aliyu et al. [51]	2018	Nigeria	*B. pfeifferi*	592	156	26.00	—	—	—	156	26.00	*S. mansoni*	8
			*B. truncatus*	5942	1421	23.90	—	—	—	1421	23.90	*S. haematobium*	
			*B. globosus*	8894	2166	24.30	—	—	—	2166	24.30	*S. haematobium*	
Afiukwa et al. [46]	2019	Nigeria	*B. globosus*	177	34	19.20	—	—	—	34	19.20	*S. haematobium*	8
			*B. truncatus*	106	18	17.00	—	—	—	18	17.00	*S. haematobium*	
Okeke et al. [45]	2019	Nigeria	*B. pfeifferi*	212	-	-	212	16	7.45	16	7.45	*S. mansoni*	8
Peletu et al. [44]	2019	Nigeria	*B. globosus*	112	5	4.50	—	—	—	5	4.50	*S. haematobium*	9
Rabone et al. [42]	2019	Niger	*B. forskalii*	11989	24	0.20	—	—	—	24	0.20	*S. haematobium*	9
			*B. pfeifferi*	2290	79	3.40	—	—	—	79	3.40	*S. mansoni*	
Tian-Bi et al. [24]	2019	Côte d'Ivoire	*B. truncatus*	1772	2	0.10	—	—	—	2	0.10	*S. haematobium*	9
			*B. globosus*	247	5	2.00	—	—	—	5	2.00	*S. haematobium*	
Catalano et al. [56]	2020	Senegal	*B. pfeifferi*	407	9	2.20	—	—	—	9	2.20	*S. mansoni*	7
Fuss et al. [59]	2020	Tanzania	*B. sudanica*	788	—	—	788	279	35.40	279	35.4	*S. mansoni*	9
Mutsaka-Makuvaza et al. [68]	2020	Zimbabwe	*B. globosus*	1542	30	1.90	—	—	—	30	1.90	*S. haematobium*	9

**Table 2:** The pooled prevalences of *S. mansoni* and *S. haematobium* infection among *Biomphalaria* and *Bulinus* snails of Africa.

Snail genus	Snail species	Studies (*n*)	Examined snails (*n*)	Infected snails	
				*n*	pp (95%CI)
*Biomphalaria*	*B. pfeifferi*	19	46480	987	5.10 (4.05–6.15)
	*B. sudanica*	5	47027	1032	6.73 (4.46–9.01)
	*B. alexandrina*	4	3569	62	2.81 (0.75–4.87)
	*B. choanomphala*	2	16,100	225	1.58 (0.44–2.21)
	*B. stanleyi*	1	21,715	949	4.37 (4.09–4.64)
	Unclassified *Biomphalaria* snails	3	20414	479	21.28 (-1.78–44.34)

*Bulinus*	*B. globosus*	14	13691	2511	12.25 (6.23–18.27)
	*B. truncatus*	13	42538	2077	5.78 (4.36–7.20)
	*B. nasutus*	2	28648	278	0.98 (0.76–1.20)
	*B. senegalensis*	2	7344	10	4.73 (-10.72–20.18)
	*B. umbilicatus*	1	339	22	6.49 (3.71–9.27)
	*B. forskalii*	3	21461	37	0.13 (0.08–0.34)
	*B. camerunensis*	1	7	4	57.14 (21.30–92.99)
	Unclassified *Bulinus* snails	1	4312	9	2.1 (0.10–3.90)

	Total	51	273 643	8682	5.51 (4.95–6.07)

## Data Availability

All the datasets are included in the manuscript.

## Abbreviations

CI: Confidence interval
I^2^: Inverse variance index
NOS: Newcastle–Ottawa quality assessment scale
PRISMA: Preferred Reporting Items for Systematic Reviews and Meta-analysis
PCR: Polymerase chain reaction
NTD: Neglected tropical disease.

## Supplementary Materials

Supplementary Materials S1 Table: PRISMA checklist. S1 Text : NEWCASTLE–OTTAWA QUALITY ASSESSMENT SCALE. S1 Fig : forest plot showing the pooled effect estimate of *S. mansoni* among *Biomphalaria* snails in Africa. S2 Fig : forest plot showing the pooled prevalence estimate of *S. haematobium* among *Bulinus* snails in Africa.

## References

[1] WHO (2020). Schistosomiasis. https://www.who.int/news-room/fact-sheets/detail/schistosomiasis.

[2] Barry M. A., Simon G. G., Mistry N. (2013). Global trends in neglected tropical disease control and elimination: impact on child health. *Archives of Disease in Childhood*.

[3] Hotez P., Keiser J., Bos R., Tanner M., Utzinger J. (2006). Schistosomiasis and water resources development: systematic review, meta-analysis, and estimates of people at risk. *The Lancet Infectious Diseases*.

[4] Inobaya M. T., Olveda R. M., Chau T. N., Olveda D. U., Ross A. G. (2014). Prevention and control of schistosomiasis: a current perspective. *Research and Reports in Tropical Medicine*.

[5] Colley D. G., Bustinduy A. L., Secor W. E., King C. H. (2014). Human schistosomiasis. *The Lancet*.

[6] Gryseels B., Polman K., Clerinx J., Kestens L. (2006). Human schistosomiasis. *The Lancet*.

[7] Blanton R. E. (2019). Population structure and dynamics of helminthic infection: schistosomiasis. *Microbiology spectrum*.

[8] Pinto-Almeida A., Mendes T., de Oliveira R. N., Corrêa S. A. P., Allegretti S. M. (2016). Morphological characteristics of *Schistosoma mansoni* PZQ-resistant and -susceptible strains are different in presence of praziquantel. *Frontier Microbiology*.

[9] Rozendaal J. A. (1997). *Vector Control: Methods for Use by Individuals and Communities: Freshwater Snails*.

[10] Brown D. S. (1994). *Freshwater Snails of Africa and Their Medical Importance*.

[11] Kane R. A., Stothard J. R., Emery A. M., Rollinson D. (2008). Molecular characterization of freshwater snails in the genus *Bulinus*: a role for barcodes?. *Parasites & Vectors*.

[12] Zhao Q. P., Jiang M. S., Littlewood D. T. J., Nie P. (2010). Distinct genetic diversity of *Oncomelania hupensis*, intermediate host of *Schistosoma japonicum* in mainland China as revealed by ITS sequences. *PLoS Neglected Tropical Diseases*.

[13] Moher D., Liberati A., Tetzlaff J., Altman D. G. (2009). Preferred reporting items for systematic reviews and meta-analyses: the PRISMA statement. *BMJ*.

[14] Wells G. A., Shea B., O’Connell D., Peterson J., Welch V. (2012). *The Newcastle-Ottawa Scale (NOS) for Assessing the Quality of Nonrandomised Studies in Meta-Analyses*.

[15] Lewis-Mikhael A.-M., Bueno-Cavanillas A., Ofir Guiron T., Olmedo-Requena R., Delgado-Rodríguez M., Jiménez-Moleón J. J. (2016). Occupational exposure to pesticides and prostate cancer: a systematic review and meta-analysis. *Occupational and Environmental Medicine*.

[16] Higgins J. P. T., Thompson S. G., Deeks J. J., Altman D. G. (2003). Measuring inconsistency in meta-analyses. *BMJ*.

[17] Melsen W. G., Bootsma M. C. J., Rovers M. M., Bonten M. J. M. (2014). The effects of clinical and statistical heterogeneity on the predictive values of results from meta-analyses. *Clinical Microbiology and Infection*.

[18] Allan F., Sousa-Figueiredo J. C., Emery A. M., Paulo R., Mirante C. (2017). Mapping freshwater snails in north-western Angola: distribution, identity and molecular diversity of medically important taxa. *Parasites & Vectors*.

[19] Ibikounlé M., Mouahid G., Sakiti N. G., Massougbodji A., Moné H. (2009). Freshwater snail diversity in Benin (West Africa) with a focus on human schistosomiasis. *Acta Tropica*.

[20] Zongo D., Kabre B. G., Dianou D., Savadogo B., Poda J. N. (2009). Importance of malacological factors in the transmission of *Schistosoma haematobium* in two dams in the province of oubritenga (burkina faso). *Research Journal of Environmental Sciences*.

[21] Gryseels B. (1991). The epidemiology of schistosomiasis in Burundi and its consequences for control. *Transactions of the Royal Society of Tropical Medicine and Hygiene*.

[22] Moser W., Greter H., Schindler C. (2014). The spatial and seasonal distribution of Bulinus truncatus, Bulinus forskalii and Biomphalaria pfeifferi, the intermediate host snails of schistosomiasis, in N’Djamena, Chad. *Geospatial Health*.

[23] Kinanpara K., Yves B. K., Félix K. K., Edia E. O., Théophile G. (2013). Freshwater snail dynamics focused on potential risk of using urine as fertilizer in Katiola, an endemic area of schistosomiasis (ivory coast; West Africa). *Journal of Entomology and Zoology Studies*.

[24] Tian-Bi Y.-N. T., Webster B., Konan C. K., Allan F., Diakité N. R. (2019). Molecular characterization and distribution of Schistosoma cercariae collected from naturally infected bulinid snails in northern and central Côte d’Ivoire. *Parasites & Vectors*.

[25] Hussein A.-N. A., Bin-Dajem S. M. (2008). Prevalence of urinary schistosomiasis and Infections withTrematode larval stages in *Bulinus truncatus* snails from Qena Upper Egypt. *Journal of Applied Sciences Research*.

[26] Lotfy W. M., Hanelt B., Mkoji G. M., Loker E. S. (2011). Genotyping natural infections of *Schistosoma mansoni* in *Biomphalaria alexandrina* from damietta, Egypt, with comparisons to natural snail infections from Kenya. *Journal of Parasitology*.

[27] Aboelhadid S. M., Thabet M., El-Basel D., Taha R. (2016). Digenetic larvae in Schistosome snails from El Fayoum, Egypt with detection of *Schistosoma mansoni* in the snail by PCR. *Journal of Parasitic Diseases*.

[28] Farghaly A., Saleh A. A., Mahdy S. (2016). Molecular approach for detecting early prepatent *Schistosoma mansoni* infection in *Biomphalaria alexandrina* snail host. *Journal of Parasitic Diseases*.

[29] Hussein A.-N. A., Rabie S. A. H. (2007). *Schistosoma mansoni* and trematode larval stages in *Biomphalaria alexandrina* in Qena Governorate, Egypt. *Journal of the Egyptian-German Society of Zoology*.

[30] King C. L., Barkat R., Monto A. S., Miller F. D., Hussein M. (1982). Prevalence and intensity of schistosoma haematobium infection in six villages of upper Egypt. *The American Journal of Tropical Medicine and Hygiene*.

[31] Alebie G., Erko B., Aemero M., Petros B. (2014). Epidemiological study on *Schistosoma mansoni* infection in sanja area, Amhara region, Ethiopia. *Parasites & Vectors*.

[32] Gashaw F., Aemero M., Legesse M., Petros B., Teklehaimanot T. (2015). Prevalence of intestinal helminth infection among school children in maksegnit and enfranz towns, northwestern Ethiopia, with emphasis on *Schistosoma mansoni* infection. *Parasites & Vectors*.

[33] Amsalu G., Mekonnen Z., Erko B. (2015). A new focus of schistosomiasis mansoni in Hayk town, northeastern Ethiopia. *BMC Research Notes*.

[34] Mengistu M., Shimelis T., Torben W., Terefe A., Kassa T. (2011). Human intestinal Schistosomiasis in communities living near three rivers of Jimma town, south western Ethiopia. *Journal of Health Science Research*.

[35] Mekonnen Z., Haileselassie H., Medhin G., Erko B., Berhe N. (2012). *Schistosomia mansoni* focus in Mekele city, northern Ethiopia. *Ethiopian Medical Journal*.

[36] Alemayehu B., Tomass Z., Wadilo F., Leja D., Liang S. (2017). Epidemiology of intestinal helminthiasis among school children with emphasis on *Schistosoma mansoni* infection in Wolaita zone, Southern Ethiopia. *BMC Public Health*.

[37] Kariuki H. C., Muchiri E. M., Clennon J. A. (2004). Distribution patterns and cercarial shedding of *Bulinus nasutus* and other snails in the Msambweni area, coast province, Kenya. *The American Journal of Tropical Medicine and Hygiene*.

[38] Opisa S., Odiere M. R., Jura W. G., Karanja D. M., Mwinzi P. N. (2011). Malacological survey and geographical distribution of vector snails for schistosomiasis within informal settlements of Kisumu City, western Kenya. *Parasites & Vectors*.

[39] Sturrock R. F., Karamsadkar S. J., Ouma J. (1979). Schistosome infection rates in field snails:Schistosoma mansoniin *Biomphalaria pfeifferi* from Kenya. *Annals of Tropical Medicine & Parasitology*.

[40] Dabo A., Durand P., Morand S. (1997). Distribution and genetic diversity of *Schistosoma haematobium* within its bulinid intermediate hosts in Mali. *Acta Tropica*.

[41] Dabo A., Diarra A. Z., Machault V., Touré O., Niambélé D. S. (2015). Urban schistosomiasis and associated determinant factors among school children in Bamako, Mali, West Africa. *Infectious Diseases of Poverty*.

[42] Rabone M., Wiethase J. H., Allan F., Gouvras A. N., Pennance T. (2019). Freshwater snails of biomedical importance in the Niger River Valley: evidence of temporal and spatial patterns in abundance, distribution and infection with *Schistosoma spp*. *Parasites & Vectors*.

[43] Labbo R., Djibrilla A., Zamanka H., Garba A., Chippaux J.-P. (2007). *Bulinus forskalii*: a new potential intermediate host for *Schistosoma haematobium* in Niger. *Transactions of the Royal Society of Tropical Medicine and Hygiene*.

[44] Peletu B. J., Ofoezie I. E., Olaniyan R. F. (2019). Prevalence of fresh water snails transmitting *Schistosoma haematobium* in aponmu-lona river basin, idanre, ondo state, Nigeria. *International Journal of Marine Biology and Research*.

[45] Okeke O. C., Akinwale O. P., Ubachukwu P. O., Gyang P. V., Henry E. U. (2019). Report of high prevalence of schistosome infection in *Biomphalaria* snails from a geographic area with no previous prevalence of human schistosomiasis in Nigeria. *Acta Tropica*.

[46] Afiukwa F. N., Nwele D. E., Uguru O. E., Ibiam G. A., Onwe C. S. (2019). Transmission dynamics of urogenital schistosomiasis in the rural community of ebonyi state, south eastern Nigeria. *Journal of Parasitology Research*.

[47] Ayanda O. I. (2009). Prevalence of snail vectors of schistosomiasis and their infection rates in two localities within Ahmadu Bello University (A.B.U.) Campus, Zaria, Kaduna State, Nigeria. *Journal of Cell and Animal Biology*.

[48] Iboh C. I., Okon O. N., Etim S. E., Ukpong I. E., Ognan E. I. (2012). Urinary schistosomiasis among kindergarten and primary school children in Okpechi community, cross river state, Nigeria: a preliminary study. *Journal of Engineering Science and Technology*.

[49] Ivoke N., Ivoke O. N., Nwani C. D (2014). Prevalence and transmission dynamics of *Schistosoma haematobium* infection in a rural community of southwestern Ebonyi State, Nigeria. *Tropical Biomedicine*.

[50] Abe E. M., Oluwole A. S., Ojo D. A., Idowu O. A., Mafiana C. F. (2012). Predicting the geo-spatial distribution of *Bulinus* snail vector of urinary schistosomiasis in Abeokuta, South Western Nigeria. *The Zoologist*.

[51] Aliyu I. W., Mao P. S., Danladi S. I. (2018). Transmission patterns among freshwater snail hosts of schistosomiasis in Bauchi area of Nigeria. *GSC Biological and Pharmaceutical Sciences*.

[52] Okeke O. C., Ubachukwu P. O. (2017). Trematode infections of the freshwater snail *Biomphalaria pfeifferi* from a south-east Nigerian community with emphasis on cercariae of Schistosoma. *Journal of Helminthology*.

[53] Akinwale O. P., Kane R. A., Rollinson D. (2011). Molecular approaches to the identification ofBulinusspecies in south-west Nigeria and observations on natural snail infections with schistosomes. *Journal of Helminthology*.

[54] Akinwale O., Oso O., Salawu O. (2015). Molecular characterisation of *Bulinus* snails-intermediate hosts of schistosomes in Ogun State, South-western Nigeria. *Folia Malacologica*.

[55] Senghor B., Diaw O. T., Doucoure S., Seye M., Talla I. (2015). Study of the snail intermediate hosts of urogenital schistosomiasis in Niakhar, region of Fatick, West central Senegal. *Parasites & Vectors*.

[56] Catalano S., Léger E., Fall C. B., Anna B., Diop S. D. (2020). Multihost transmission of *Schistosoma mansoni* in Senegal, 2015–2018. *Emerging Infectious Diseases*.

[57] Mohammed N. A. I., Madsen H., Ahmed A. A. A. R. M. (2016). Types of trematodes infecting freshwater snails found in irrigation canals in the East Nile locality, Khartoum, Sudan. *Infectious Diseases of Poverty*.

[58] Getaneh A., Ashenafi A., Zerihun Y. (2019). Current status of intestinal parasitic infections and associated factors among primary school children in Birbir town, Southern Ethiopia. *BMC Infectious Diseases*.

[59] Fuss A., Mazigo H. D., Mueller A. (2020). Malacological survey to identify transmission sites for intestinal schistosomiasis on Ijinga Island, Mwanza, north-western Tanzania. *Acta Tropica*.

[60] Lwambo N. J. S. (1988). Transmission of urinary schistosomiasis in Sukumaland, Tanzania. 1 snail infection rates and incidence of infection in school children. *Journal of Helminthology*.

[61] Gouvras A. N., Allan F., Kinung’hi S., Rabone M., Emery A. (2017). Longitudinal survey on the distribution of *Biomphalaria sudanica* and *B. choanomophala* in Mwanza region, on the shores of Lake Victoria, Tanzania: implications for schistosomiasis transmission and control. *Parasites & Vectors*.

[62] Angelo T., Shahada F., Kassuku A., Mazigo H., Kariuki C. (2014). Population abundance and disease transmission potential of snail intermediate hosts of human schistosomiasis in fishing communities of Mwanza region, North-western Tanzania. *International Journal of Science and Research*.

[63] Pennance T., Person B., Muhsin M. A., Khamis A. N., Muhsin J. (2016). Urogenital schistosomiasis transmission on Unguja Island, Zanzibar: characterisation of persistent hot-spots. *Parasites & Vectors*.

[64] Odongo-Aginya E. I., Kironde F. K., Kabatereine N. B., Kategere P., Kazibwe F. (2008). Effect of seasonal rainfall and other environmental changes, on snail density and infection rates with *Schistosoma mansoni* fifteen years after the last snails’ study in Kigungu, Entebbe, Uganda. *East African medical journal*.

[65] Stanton M. C., Adriko M., Arinaitwe M., Howell A., Davies J. (2017). Intestinal schistosomiasis in Uganda at high altitude (>1400 m): malacological and epidemiological surveys on mount elgon and in fort portal crater lakes reveal extra preventive chemotherapy needs. *Infectious Diseases of Poverty Journal*.

[66] Kazibwe F., Makanga B., Rubaire-Akiiki C. (2006). Ecology of *Biomphalaria* (Gastropoda: *Planorbidae*) in Lake Albert, Western Uganda: snail distributions, infection with schistosomes and temporal associations with environmental dynamics. *Hydrobiologia*.

[67] Rowel C., Fred B., Betson M., Sousa-Figueiredo J. C., Kabatereine N. B. (2015). Environmental epidemiology of intestinal schistosomiasis in Uganda: population dynamics of *Biomphalaria* (gastropoda: Planorbidae) in lake albert and Lake Victoria with observations on natural infections with digenetic trematodes. *BioMed Research International*.

[68] Mutsaka-Makuvaza M. J., Zhou X.-N., Tshuma C., Abe E., Manasa J. (2020). Molecular diversity of *Bulinus* species in Madziwa area, Shamva district in Zimbabwe: implications for urogenital schistosomiasis transmission. *Parasites & Vectors*.

[69] Bakuza J. S., Gillespie R., Nkwengulila G. (2017). Assessing *S. mansoni* prevalence in *Biomphalaria* snails in the Gombe ecosystem of western Tanzania: the importance of DNA sequence data for clarifying species identification. *Parasites and Vectors*.

[70] Sokolow S. H., Wood C. L., Jones I. J. (2018). To reduce the global burden of human schistosomiasis, use ’old fashioned’ snail control. *Trends in Parasitology*.

[71] Tanaka H., Tsuji M. (1997). From discovery to eradication of schistosomiasis in Japan: 1847-1996. *International Journal for Parasitology*.

[72] Utzinger J., Raso G., Brooker S. (2009). Schistosomiasis and neglected tropical diseases: towards integrated and sustainable control and a word of caution. *Parasitology*.

[73] Adenowo A. F., Oyinloye B. E., Ogunyinka B. I., Kappo A. P. (2015). Impact of human schistosomiasis in sub-Saharan Africa. *The Brazilian Journal of Infectious Diseases*.

[74] Barakat R., El Morshedy H., Farghaly A., McDowell M. A., Rafati S. (2014). Human schistosomiasis in the Middle East and north Africa region. *Neglected Tropical Diseases - Middle East and North Africa*.

[75] Calasans T. A. S., Souza G. T. R., Melo C. M., Madi R. R., Jeraldo V. S. (2018). Socioenvironmental factors associated with *Schistosoma mansoni* infection and intermediate hosts in an urban area of northeastern Brazil. *PLoS One*.

[76] Satrija F., Ridwan Y., Jastal S., Samarang A., Rauf A. (2015). Current status of schistosomiasis in Indonesia. *Acta Tropica*.

[77] Gandasegui J., Fernández-Soto P., Muro A., Simões Barbosa C., Lopes de Melo F. (2018). A field survey using LAMP assay for detection of *Schistosoma manson*i in a low-transmission area of schistosomiasis in Umbuzeiro, Brazil: assessment in human and snail samples. *PLoS Neglected Tropical Diseases*.

[78] Worku L., Damte D., Endris M., Tesfa H., Aemero M. (2014). *Schistosoma mansoni* infection and associated determinant factors among school children in sanja town, northwest Ethiopia. *Journal of Parasitology Research*.

[79] Akinwale O. P., Ajayi M. B., Akande D. O., Adeleke M. A., Gyang P. V. (2009). Prevalence of *Schistosoma haematobium* infection in a neglected community, south western Nigeria. *International Healthcare Research Journal*.

[80] Ezeh C. O., Onyekwelu K. C., Akinwale O. P., Shan L., Wei H. (2019). Urinary schistosomiasis in Nigeria: a 50 year review of prevalence, distribution and disease burden. *Parasite*.

[81] Abe E. M., Guan W., Guo Y.-H., Kassegne K., Qin Z.-Q. (2018). Differentiating snail intermediate hosts of *Schistosoma* spp. using molecular approaches: fundamental to successful integrated control mechanism in Africa. *Infectious Diseases of Poverty*.

[82] Dennis E., Vorkpor P., Holzer B. (1983). Studies on the epidemiology of schistosomiasis in Liberia: the prevalence and intensity of schistosomal infections in Bong County and the bionomics of the snail intermediate hosts. *Acta Tropica*.

[83] Diakité N. R., Winkler M. S., Coulibaly J. T., Guindo-Coulibaly N., Utzinger J. (2017). Dynamics of freshwater snails and *Schistosoma* infection prevalence in schoolchildren during the construction and operation of a multipurpose dam in central Côte d’Ivoire. *Infectious Diseases of Poverty*.

[84] Picquet M., Ernould J. C., Vercruysse J. (1996). The epidemiology of human schistosomiasis in the Senegal river basin. *Transactions of the Royal Society of Tropical Medicine and Hygiene*.

[85] Kock K., Wolmarans C., Bornman M. (2004). Distribution and habitats of *Biomphalaria pfeifferi,* snail intermediate host of *Schistosoma mansoni*, in South Africa. *Water SA*.

[86] Kengne-Fokam A. C., Nana-Djeunga H. C., Bagayan M., Njiokou F. (2018). *Biomphalaria camerunensis* as a viable alternative intermediate host for *Schistosoma mansoni* in southern Cameroon. *Parasites & Vectors*.

[87] Mitiku H., Legesse M., Teklemariam Z., Erko B. (2011). Transmission of *Schistosoma mansoni* in tikur wuha area, southern Ethiopia. *The Ethiopian Journal of Health Development*.

[88] Mutuku M. W., Lu L., Otiato F. O. (2017). A comparison of Kenyan *Biomphalaria pfeifferi* and *B. Sudanica* as vectors for *Schistosoma mansoni,* including a discussion of the need to better understand the effects of snail breeding systems on transmission. *Journal of Parasitology*.

[89] Gabrielli A. F., Ramsan M., Naumann C. (2005). Soil-transmitted helminths and haemoglobin status among Afghan children in world food programme assisted schools. *Journal of Helminthology*.

[90] Erko B., Balcha F., Kifle D. (2006). The ecology of *Biomphalaria sudanica* in Lake Ziway, Ethiopia. *African Journal of Ecology*.

[91] Abou-El-Naga I. F. (2013). *Biomphalaria alexandrina* in Egypt: past, present and future. *Journal of Biosciences*.

[92] El-Nassery S. M. F., Abou-El-Naga I. F., Allam S. R., Shaat E. A., Mady R. F. M. (2013). Genetic variation between *Biomphalaria alexandrina* snails susceptible and resistant to *schistosoma manson*i infection. *BioMed Research International*.

[93] Mansour T. A., Habib M. R., Rodríguez L. C. V., Vázquez A. H., Alers J. M. (2017). Central nervous system transcriptome of *Biomphalaria alexandrina*, an intermediate host for schistosomiasis. *BMC Research Notes*.

[94] Bandoni S. M., Mulvey M., Loker E. S. (2000). Population structure and taxonomic discrimination among three species of *Biomphalaria preston*, 1910 (Gastropoda: *Planorbidae*) from Kenya. *Zoological Journal of the Linnean Society*.

[95] Abdulkadir A., Ahmed M., Abubakar B. M. (2017). Prevalence of urinary schistosomiasis in Nigeria, 1994-2015: systematic review and meta-analysis. *African Journal of Urology*.

[96] Odeniran P. O., Omolabi K. F., Ademola I. O. (2020). Epidemiological dynamics and associated risk factors of S. haematobium in humans and its snail vectors in Nigeria: a meta-analysis (1983-2018). *Pathogens and Global Health*.

[97] Hamburger J., Hoffman O., Kariuki H. C. (2004). Large-scale, polymerase Chain reaction-based surveillance of schistosoma haematobium dna in snails from transmission sites in coastal Kenya: a new tool for studying the dynamics of snail infection. *The American Journal of Tropical Medicine and Hygiene*.

[98] Abath F. G., Gomes A. L. d. V., Melo F. L., Barbosa C. S., Werkhauser R. P. (2006). Molecular approaches for the detection of *Schistosoma manson*i: possible applications in the detection of snail infection, monitoring of transmission sites, and diagnosis of human infection. *Memórias Do Instituto Oswaldo Cruz*.

[99] Caldeira R. L., Jannotti-Passos L. K., Dos Santos Carvalho O. (2017). Use of molecular methods for the rapid mass detection of *Schistosoma mansoni* (Platyhelminthes: trematoda) in *Biomphalaria* spp. (gastropoda: Planorbidae). *Journal of Tropical Medicine*.

[100] Gandasegui J., Fernández-Soto P., Hernández-Goenaga J., López-Abán J., Vicente B. (2016). Biompha-LAMP: a new rapid loop-mediated isothermal amplification assay for detecting *Schistosoma mansoni* in *Biomphalaria glabrata* snail host. *PLoS Negl Trop Dis*.

