# Supplementary material for: Prevalence of Schistosoma mansoni and S. haematobium in Snail Intermediate Hosts in Africa: A Systematic Review and Meta-analysis
Source: J Trop Med. 2020 Sep 7;2020:8850840. doi: 10.1155/2020/8850840 (PMC7492904; doi:10.1155/2020/8850840)
Supplement: Supplementary Materials — S1 Table: PRISMA checklist. S1 Text : NEWCASTLE–OTTAWA QUALITY ASSESSMENT SCALE. S1 Fig : forest plot showing the pooled effect estimate of S. mansoni among Biomphalaria snails in Africa. S2 Fig : forest plot showing the pooled prevalence estimate of S. haematobium among Bulinus snails in Africa. [file 8850840.f1.doc]

**S1 Text: NEWCASTLE - OTTAWA QUALITY ASSESSMENT SCALE**

(Adapted for crossectional studies)

**Selection: (Maximum 5 stars)**

**1) Representativeness of the sample:**

1. Truly representative of the average in the target population. * (all subjects or random sampling)
2. Somewhat representative of the average in the target population. * (non-random sampling)
3. Selected group of users.
4. No description of the sampling strategy.

**2) Sample size:**

1. Justified and satisfactory. *
2. Not justified.

**3) Non-respondents:**

1. Comparability between respondents and non-respondents characteristics is established, and the response rate is satisfactory. *
2. The response rate is unsatisfactory, or the comparability between respondents and non-respondents is unsatisfactory.
3. No description of the response rate or the characteristics of the responders and the non-responders.

**4) Ascertainment of the exposure (risk factor):**

1. Validated measurement tool. **
2. Non-validated measurement tool, but the tool is available or described*
3. No description of the measurement tool.

**Comparability: (Maximum 2 stars)**

1) The subjects in different outcome groups are comparable, based on the study design or analysis. Confounding factors are controlled.

1. The study controls for the most important factor (select one). *
2. The study control for any additional factor. *

**Outcome: (Maximum 3 stars)**

**1) Assessment of the outcome:**

1. Independent blind assessment. **
2. Record linkage. **
3. Self report.
4. No description.

2) Statistical test:

1. The statistical test used to analyze the data is clearly described and appropriate, and the measurement of the association is presented, including confidence intervals and the probability level *(p-value*). *
2. The statistical test is not appropriate, not described or incomplete.

This scale has been adapted from the Newcastle-Ottawa Quality Assessment Scale for cohort studies to perform a quality assessment of cross-sectional studies for the systematic review, “Are Healthcare Workers’ Intentions to Vaccinate Related to their Knowledge, Beliefs and Attitudes? A Systematic Review”.

We have not selected one factor that is the most important for comparability, because the variables are not the same in each study. Thus, the principal factor should be identified for each study.

In our scale, we have specifically assigned one star for self-reported outcomes, because our study measures the intention to vaccinate. Two stars are given to the studies that assess the outcome with independent blind observers or with vaccination records, because these methods measure the practice of vaccination, which is the result of true intention.

**S1 Table** PRISMA Checklist

| **Section/topic** | **#** | **Checklist item** | | | **Reported on page #** | |
| --- | --- | --- | --- | --- | --- | --- |
| **TITLE** | | | | |  | |
| Title | 1 | | Identify the report as a systematic review, meta-analysis, or **both**. | | Title page | |
| **ABSTRACT** | | | | |  | |
| Structured summary | 2 | | Provide a structured summary including, as applicable: background; objectives; data sources; study eligibility criteria, participants, and interventions; study appraisal and synthesis methods; results; limitations; conclusions and implications of key findings; systematic review registration number. | | Abstract (structured) | |
| **INTRODUCTION** | | | | |  | |
| Rationale | 3 | | Describe the rationale for the review in the context of what is already known. | | Introduction, paragraphs 1-5 | |
| Objectives | 4 | | Provide an explicit statement of questions being addressed with reference to participants, interventions, comparisons, outcomes, and study design (PICOS). | | Introduction, paragraph 5 | |
| **METHODS** | | | | |  | |
| Protocol and registration | 5 | | Indicate if a review protocol exists, if and where it can be accessed (e.g., Web address), and, if available, provide registration information including registration number. | | - | |
| Eligibility criteria | 6 | | Specify study characteristics (e.g., PICOS, length of follow-up) and report characteristics (e.g., years considered, language, publication status) used as criteria for eligibility, giving rationale. | | Methods, paragraphs 1-3 | |
| Information sources | 7 | | Describe all information sources (e.g., databases with dates of coverage, contact with study authors to identify additional studies) in the search and date last searched. | | Methods, paragraph 1 | |
| Search | 8 | | Present full electronic search strategy for at least one database, including any limits used, such that it could be repeated. | | Methods, paragraph 1 | |
| Study selection | 9 | | State the process for selecting studies (i.e., screening, eligibility, included in systematic review, and, if applicable, included in the meta-analysis). | | Methods, paragraph 1&2 | |
| Data collection process | 10 | | Describe method of data extraction from reports (e.g., piloted forms, independently, in duplicate) and any processes for obtaining and confirming data from investigators. | | Methods, paragraph 3 | |
| Data items | 11 | | List and define all variables for which data were sought (e.g., PICOS, funding sources) and any assumptions and simplifications made. | | Methods, paragraph 6 | |
| Risk of bias in individual studies | 12 | | Describe methods used for assessing risk of bias of individual studies (including specification of whether this was done at the study or outcome level), and how this information is to be used in any data synthesis. | | Methods, paragraph 4 | |
| Summary measures | 13 | | State the principal summary measures (e.g., risk ratio, difference in means). | | Methods, paragraph 6 | |
| Synthesis of results | 14 | | Describe the methods of handling data and combining results of studies, if done, including measures of consistency (e.g., I^2^) for each meta-analysis. | | Methods, paragraph 6 | |
| **Section/topic** | **#** | | **Checklist item** | | | **Reported on page #** |
| Risk of bias across studies | 15 | | Specify any assessment of risk of bias that may affect the cumulative evidence (e.g., publication bias, selective reporting within studies). | | | Methods, paragraph 5 |
| Additional analyses | 16 | | Describe methods of additional analyses (e.g., sensitivity or subgroup analyses, meta-regression), if done, indicating which were pre-specified. | | | Methods, paragraph 6 |
| **RESULTS** | | | | | |  |
| Study selection | 17 | | | Give numbers of studies screened, assessed for eligibility, and included in the review, with reasons for exclusions at each stage, ideally with a flow diagram. | | Results, paragraph 1 (Fig. 1) |
| Study characteristics | 18 | | | For each study, present characteristics for which data were extracted (e.g., study size, PICOS, follow-up period) and provide the citations. | | Results, paragraph 2 (Table 1) |
| Risk of bias within studies | 19 | | | Present data on risk of bias of each study and, if available, any outcome level assessment (see item 12). | | Results, paragraph 3 & Table-1 Quality S |
| Results of individual studies | 20 | | | For all outcomes considered (benefits or harms), present, for each study: (a) simple summary data for each intervention group (b) effect estimates and confidence intervals, ideally with a forest plot. | | Results, paragraph 3, Table 2 & Figure 2,4,5,&6 |
| Synthesis of results | 21 | | | Present results of each meta-analysis done, including confidence intervals and measures of consistency. | | Results, paragraph 4 & Figure 2,4,5 & 6 |
| Risk of bias across studies | 22 | | | Present results of any assessment of risk of bias across studies (see Item 15). | | Results, paragraph 10 & Fig. 7 |
| Additional analysis | 23 | | | Give results of additional analyses, if done (e.g., sensitivity or subgroup analyses, meta-regression [see Item 16]). | | Results, paragraph 5-9,11, Fig 3-6, S1-2 Fig |
| **DISCUSSION** | | | | | |  |
| Summary of evidence | 24 | | | Summarize the main findings including the strength of evidence for each main outcome; consider their relevance to key groups (e.g., healthcare providers, users, and policy makers). | | Discussion, paragraphs 1-14 |
| Limitations | 25 | | | Discuss limitations at study and outcome level (e.g., risk of bias), and at review-level (e.g., incomplete retrieval of identified research, reporting bias). | | Discussion, paragraph 15 |
| Conclusions | 26 | | | Provide a general interpretation of the results in the context of other evidence, and implications for future research. | | Conclusions |
| **FUNDING** | | | | | |  |
| Funding | 27 | | | Describe sources of funding for the systematic review and other support (e.g., supply of data); role of funders for the systematic review. | | Not available |

**S1 Figure** Forest plot showing the pooled effect estimate of *S. mansoni* among *Biomphalaria* snails in Africa

**S2 Figure** Forest plot showing the pooled prevalence estimate of *S. haematobium* among *Bulinus* snails in Africa
